# Harmonic Aggregation Entropy: A Highly Discriminative Harmonic Feature Estimator for Time Series

**DOI:** 10.3390/e27070738

**Published:** 2025-07-10

**Authors:** Ye Wang, Zhentao Yu, Cheng Chi, Bozhong Lei, Jianxin Pei, Dan Wang

**Affiliations:** 1 Naval Submarine Academy, Qingdao 266199, China; wangye2022@foxmail.com (Y.W.); qyyzt@163.com (Z.Y.); cheng.chihhu@163.com (C.C.); leibozhong2023@foxmail.com (B.L.); 2 College of Marine Geoscience, Ocean University of China, Qingdao 266100, China; peijx@ouc.edu.cn

**Keywords:** harmonic detection, bispectrum integration, entropy feature, shaft-rate magnetic field

## Abstract

Harmonics are a common phenomenon widely present in power systems. The presence of harmonics not only increases the energy consumption of equipment but also poses hidden risks to the safety and stealth performance of large ships. Thus, there is an urgent need for a detection method for the harmonic characteristics of time series. We propose a novel harmonic feature estimation method, termed Harmonic Aggregation Entropy (HaAgEn), which effectively discriminates against background noise. The method is based on bispectrum analysis; utilizing the distribution characteristics of harmonic signals in the bispectrum matrix, a new Diagonal Bi-directional Integral Bispectrum (DBIB) method is employed to effectively extract harmonic features within the bispectrum matrix. This approach addresses the issues associated with traditional time–frequency analysis methods, such as the large computational burden and lack of specificity in feature extraction. The integration results, $I_{x}$ and $I_{y}$, of DBIB on different frequency axes are calculated using cross-entropy to derive HaAgEn. It is verified that HaAgEn is significantly more sensitive to harmonic components in the signal compared to other types of entropy, thereby better addressing harmonic detection issues and reducing feature redundancy. The detection accuracy of harmonic components in the shaft-rate electromagnetic field signal, as evidenced by sea trial data, reaches 96.8%, which is significantly higher than that of other detection methods. This provides a novel technical approach for addressing the issue of harmonic detection in industrial applications.

## 1. Introduction

In industrial and power systems, harmonic detection is widely used in power quality analysis, equipment condition monitoring, and fault diagnosis. Non-linear loads in power systems, such as frequency converters and rectifiers, introduce harmonic components, leading to grid pollution and equipment losses [1]. Accurate harmonic detection and analysis can effectively evaluate power quality, optimize the operational efficiency of power systems, and prevent potential equipment failures. Moreover, harmonic detection technology demonstrates unique application potential in the field of underwater detection. Modern ships and underwater vehicles frequently house large electrical devices that generate complex electromagnetic signals during operation. Specifically, during propeller propulsion, the current from the power system forms a loop in the seawater along the propeller shaft structure, thereby radiating extremely low-frequency electromagnetic fields, also known as shaft-rate electromagnetic fields [2]. This electromagnetic signal exhibits typical harmonic characteristics, with its frequency components closely related to the propeller’s speed and the power system’s operational state [3]. By analyzing harmonic components of electromagnetic signals in seawater, the detection, identification, and tracking of underwater targets can be achieved, offering significant technical support for the protection of marine safety.

Seawater, as a complex conductive medium, significantly attenuates and interferes with the propagation of electromagnetic signals, posing great challenges for the practical application of harmonic detection technology. Traditional harmonic analysis methods often struggle to accurately extract the characteristics of the target signal in low-signal-to-noise-ratio (SNR) environments [4,5,6,7]. Therefore, the development of high-precision and high-robustness harmonic detection algorithms has become a key focus of current research [8,9]. In recent years, harmonic detection methods based on time–frequency analysis, machine learning, and information entropy theory have gradually gained attention [10,11,12,13]. Zhu [14] proposed a rapid harmonic detection method based on an extended input observer, which constructs a state-space model based on the original signal and harmonic components and uses the input observer to estimate state variables, thereby achieving rapid harmonic extraction. Even under different signal-to-noise ratio (SNR) white noise interferences, this method can still quickly and accurately complete harmonic detection, showing strong anti-interference capability. Zhang [15] combined the S transform and bispectral analysis to propose a new STB method, which has good multiscale time–frequency focusing performance and can retain the absolute phase characteristics of the signal; bispectral analysis can suppress Gaussian noise, preserve phase information, and quantitatively describe the quadratic phase coupling in the signal. Wilkinson [16] introduced the discrete wavelet transform into transient harmonic detection in power systems, achieving rapid localization and suppression of high-frequency components with certain multiresolution advantages. Lu [17] proposed an active filter harmonic current detection algorithm based on generalized delayed signal superposition, addressing the poor filtering performance and adaptability to frequency variations of traditional harmonic detection methods in variable frequency power grids. Experimental validation under various complex conditions demonstrated the superior detection accuracy and robustness of the method. Researchers [18,19,20,21] have used machine learning for harmonic detection and suppression, which has improved the accuracy and adaptability of harmonic detection to some extent, but the input of time–frequency graphs or signal multicomponents increases computational complexity.

Bispectral analysis offers unique advantages in harmonic detection, enabling the calculation of phase relationships between frequency components and the identification of frequency coupling effects in non-linear systems. Mohebbi [22] captures non-linear interactions in signals by combining spectral and bispectral features, significantly improving prediction sensitivity and specificity; however, the model’s generalization ability is limited. Saidi [23] proposed a deterministic bispectral analysis method to extract phase coupling characteristics of coupled harmonic random signals, applied to rotor fault diagnosis. By suppressing Gaussian noise interference, high-precision identification of bearing cracks and unbalanced faults was achieved. The wavelet bispectrum, which combines the time–frequency resolution of the wavelet transform with the high-order statistical properties of the bispectrum, was used to analyze the non-linear characteristics of non-stationary signals. Newman [24] defined the wavelet bispectrum. By combining the time–frequency resolution of the wavelet transform with the high-order statistical characteristics of the bispectrum, the transform was used to analyze the non-linear characteristics of nonstationary signals, verifying the detection ability of instantaneous phase coupling. Li [25] used bispectral analysis to detect the non-linear dynamic response of beam structures with breathing cracks, identifying crack position and depth through bispectral amplitude and phase information, revealing non-linear harmonic coupling caused by the crack opening and closing effects. Jaakko [26] applied bispectrum estimation to the time–frequency analysis of ground surveillance radar echo signals, extracting target micromotion characteristics through the bicoherence coefficient. Compared to short-time Fourier transform, bispectrum offers greater advantages in suppressing cross-term interference [27]. Although bispectral analysis has been applied as a signal detection method across numerous domains, its generic application using raw bispectral images as input often lacks specificity due to the distinct requirements of different scenarios. Therefore, research should focus on developing scenario-specific solutions based on bispectral analysis. In the context of detecting ship-radiated shaft-rate magnetic field signals underwater, it is particularly crucial to extract highly targeted features from vast volumes of marine monitoring data.

This paper proposes a harmonic feature estimation method for time series based on harmonic aggregation entropy, addressing the challenge of harmonic detection in complex environments. First, phase coupling and spectral characteristics of harmonic components are extracted using bisspectral calculation, and a new bispectral integration method (DBIB) is proposed, which compresses harmonic features into two one-dimensional integrals for feature dimension reduction. This improved characterization of the harmonic components in the signal provides input for subsequent deep learning and other detection methods. A new entropy measure, HaAgEn, is proposed, which calculates the cross-entropy of bispectral integration results to estimate the consistency of the signal’s frequency distribution across different integration paths, reducing feature redundancy and enhancing the relevance of harmonic feature detection. The method is verified using harmonic signals measured during sea trials.The main contributions of this paper are as follows:

(1) A new bispectral integration method is proposed that utilizes the frequency coherence of harmonics to project features onto different frequency axes and addresses the issues of complex computation and feature redundancy in directly extracting features from the bispectrum matrix.
(2) Based on DBIB, a new entropy theory is proposed to evaluate the consistency of integration results across two frequency axes by calculating their cross-entropy. The stronger the consistency, the smaller the entropy value, making the harmonics more prominent. Compared with other entropy measures, this method demonstrates greater discrimination and sensitivity to harmonics.
(3) HaAgEn is combined with a convolutional neural network to verify its effectiveness in the harmonic signal measured in the sea trial. Compared with other harmonic detection methods, the detection accuracy of the proposed method is significantly improved, reaching 96.8%.

This paper is structured as follows: Section 2 introduces the theoretical foundations of the proposed method; Section 3 verifies the method’s superiority through comparative experiments using synthetic signals; Section 4 demonstrates its enhanced performance in real-world scenarios using sea trial datasets; and Section 5 summarizes the study and discusses potential directions for future research.

## 2. Methodology

### 2.1. Bispectrum Analysis

In traditional signal processing, the Fourier transform and power spectrum are primarily used to analyze the linear characteristics and energy distribution of signals [28]. However, these methods are limited to revealing second-order correlation information and are ineffective in capturing non-linear structures, harmonic coupling, and higher-order statistical features within signals. To address these limitations, scientists introduced higher-order spectral analysis in the mid-20th century [29], with the bispectrum emerging as one of its most significant tools.

The bispectrum is a specific implementation of the third-order spectrum. By performing a two-dimensional Fourier transform on the third-order cumulant of a signal, it captures the interactions and phase coupling among three frequency components. Unlike the power spectrum, which only reflects the energy of frequency components, the bispectrum can reveal non-linear characteristics, non-Gaussianity, and the coupling between harmonics [30,31]. Therefore, the bispectrum offers unique advantages in areas such as non-linear system detection, harmonic analysis, and fault diagnosis [32].

It is known that for a discrete-time sequence x(t) of length *N*, its DFT (Discrete Fourier Transform) is given by the following:

(1) $$X\left(f\right)=\sum_{t=0}^{N-1}x\left(t\right)e^{-j2\pi ft/N}$$

According to the definition of higher-order spectra, the bispectrum is the third-order cumulant, which corresponds to the two-dimensional Fourier transform of x(t):

(2) $$B_{x}\left(f_{1},f_{2}\right)=E\left[X\left(f_{1}\right)X\left(f_{2}\right)X^{*}\left(f_{1}+f_{2}\right)\right]$$

- E[·] Represents the mathematical expectation, which is typically replaced by time averaging or segment averaging in practical applications.
- $X^{*}$ Represents the conjugate of *X*.

Expanding yields the following result, assuming x(t) has zero mean:

(3) $$\begin{array}{cc} B_{x}\left(f_{1},f_{2}\right) & =E\left[\left(\sum_{t_{1}}x\left(t_{1}\right)e^{-j2\pi f_{1}t_{1}/N}\right)\left(\sum_{t_{2}}x\left(t_{2}\right)e^{-j2\pi f_{2}t_{2}/N}\right)\left(\sum_{t_{3}}x^{*}\left(t_{3}\right)e^{j2\pi \left(f_{1}+f_{2}\right)t_{3}/N}\right)\right]\\ \; & =E\left[\sum_{t_{1}}\sum_{t_{2}}\sum_{t_{3}}x\left(t_{1}\right)x\left(t_{2}\right)x^{*}\left(t_{3}\right)e^{-j2\pi (f_{1}t_{1}+f_{2}t_{2}-\left(f_{1}+f_{2}\right)t_{3})/N}\right] \end{array}$$

If x(t) is a wide-sense stationary process, $E[x\left(t_{1}\right)x\left(t_{2}\right)x^{*}\left(t_{3}\right)]$ depends only on the time differences $\left(t_{1}-t_{3},t_{2}-t_{3}\right)$, denoted as $C_{3}\left(\tau_{1},\tau_{2}\right)$.

(4) $$B_{x}\left(f_{1},f_{2}\right)=\sum_{\tau_{1}}\sum_{\tau_{2}}C_{3}\left(\tau_{1},\tau_{2}\right)e^{-j2\pi (f_{1}\tau_{1}+f_{2}\tau_{2})}$$

When harmonic components are present in a signal, assuming that the fundamental frequency is $f_{0}$, its harmonics are integer multiples of the fundamental, such as $f_{1}=k_{1}f_{0}$ and $f_{2}=k_{2}f_{0}$. This also implies the existence of phase coupling and non-linear relationships between frequencies (for example, $f_{3}=f_{1}+f_{2}$).

When computing the DFT, X(f) is nonzero only at frequencies $f=kf_{0}$. In contrast, bispectrum analysis essentially examines whether the phases of $f_{1},f_{2}$, and $f_{1}+f_{2}$ are correlated. For the aforementioned harmonic signal, $X(f_{1})$, $f_{2}$, and $X^{*}\left(f_{1}+f_{2}\right)$ are all nonzero at the harmonic frequencies, and the sum of their phases is

(5) $$\phi_{f_{1}}+\phi_{f_{2}}-\phi_{f_{1}+f_{2}}$$

If there is a definite phase relationship between the harmonics (for example, orderly harmonics or non-linear coupling), the phases will not be random. Consequently, the expectation of the product of the three components will be nonzero, and the bispectrum will display significant values at these points. For Gaussian noise or signals without harmonics, the frequency components are completely independent and their phases are random, resulting in $E\left[X\left(f_{1}\right)X\left(f_{2}\right)X^{*}\left(f_{1}+f_{2}\right)\right]=0$, and a bispectrum that is zero or negligible.

### 2.2. Integrated Bispectrum

The bispectrum is a two-dimensional function that can be directly input into a convolutional neural network (CNN) for classification [33,34]. However, the application of the bispectrum in fusion is significantly constrained due to the substantial computational requirements. Consequently, bispectrum values can be integrated based on specific criteria, allowing the transformation of this two-dimensional function into a one-dimensional representation [35], which significantly alleviates the classification difficulty and reduces computational load. Additionally, leveraging the symmetry of the bispectrum further decreases the computational burden.

**Figure 1:**
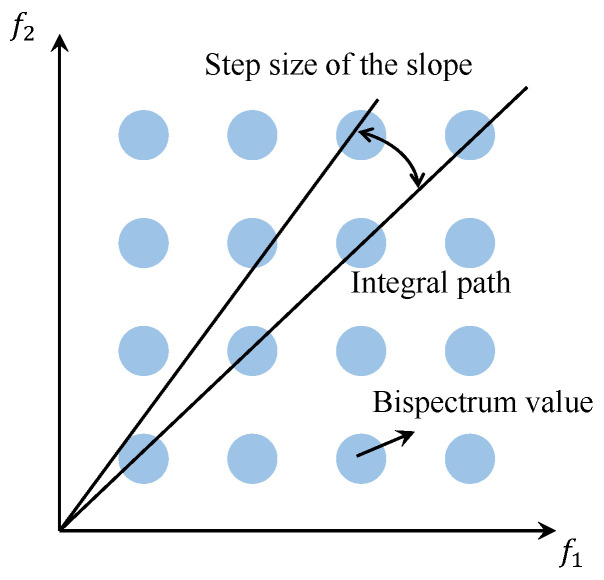
RIB schematic diagram.

As illustrated in Figure 1, within the bispectrum plane, the integral path follows a straight line passing through the origin, resulting in the value known as the Radially Integrated Bispectrum (RIB). The RIB presents a significant drawback: if the slope step size is selected too large, even if all $f_{1}$ points along the integration path are rounded, certain points may still be overlooked, potentially containing important information. Conversely, if the slope step size is chosen too small, the rounded $f_{2}$ points along the path will inevitably be repeated, leading to the repeated integration of certain bispectrum values and resulting in less distinct characteristics of the bispectrum integral values.

**Figure 2:**
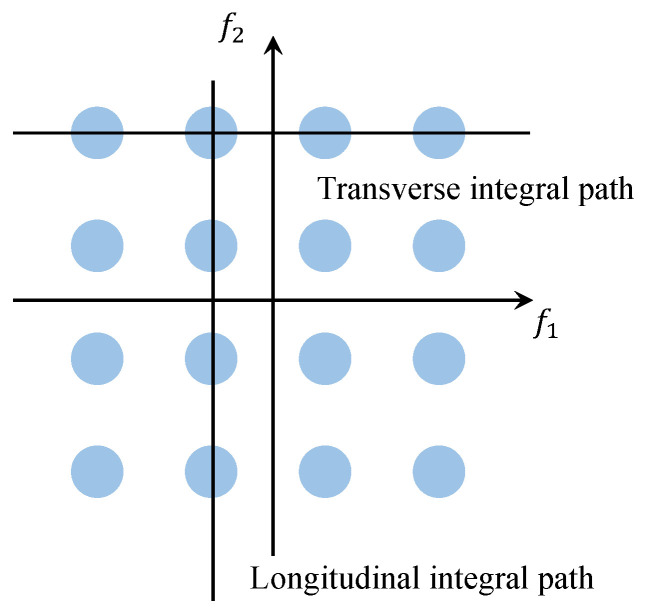
AIB schematic diagram.

As illustrated in Figure 2, within the bispectrum plane, the integration path follows a line that is either parallel to the $f_{1}$ axis or parallel to the $f_{2}$ axis, yielding the value known as the Axially Integrated Bispectrum (AIB). The AIB incorporates every bispectrum value and guarantees that these values are not reused; however, it results in a significant loss of phase information.

**Figure 3:**
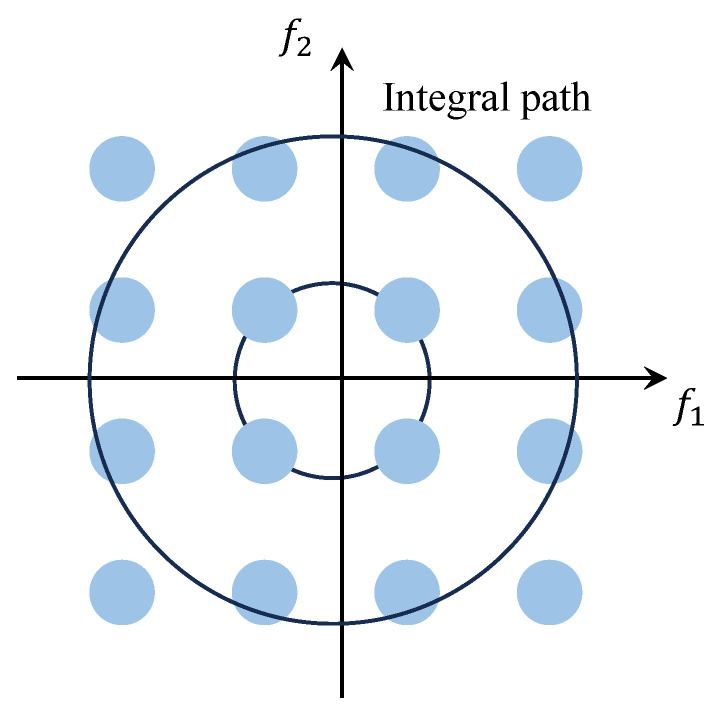
CIB schematic diagram.

As illustrated in Figure 3, within the bispectrum plane, the integration path follows a circle centered at the origin, yielding the value known as the Circularly Integrated Bispectrum (CIB). Similarly, the CIB is likely to overlook certain bispectrum values during integration due to the step size.

**Figure 4:**
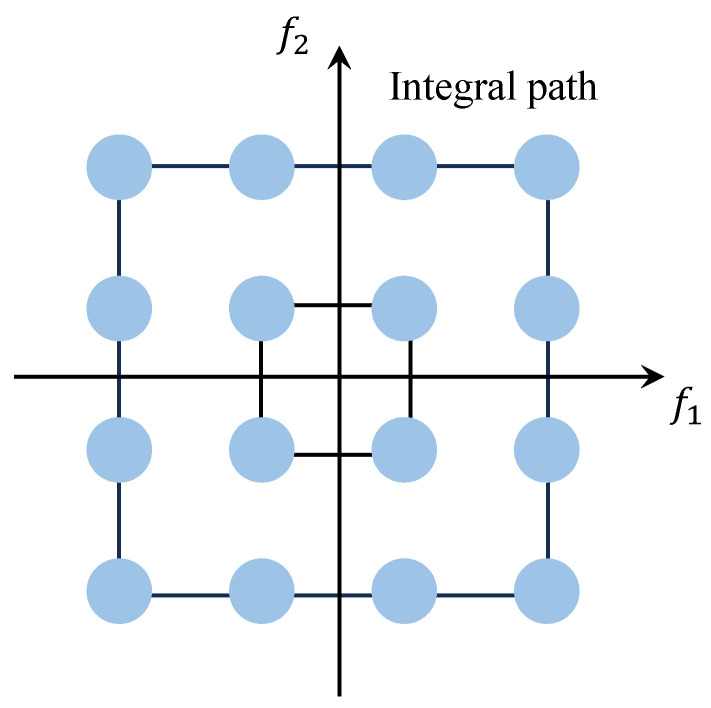
SIB schematic diagram.

As illustrated in Figure 4, within the bispectrum plane, the integration path follows a square centered at the origin, yielding the value known as the Surround-Line Integral Bispectrum (SIB). The SIB utilizes each bispectral value and guarantees that these values are not reused, while also preserving the scale information and a portion of the phase information of the signal.

**Figure 5:**
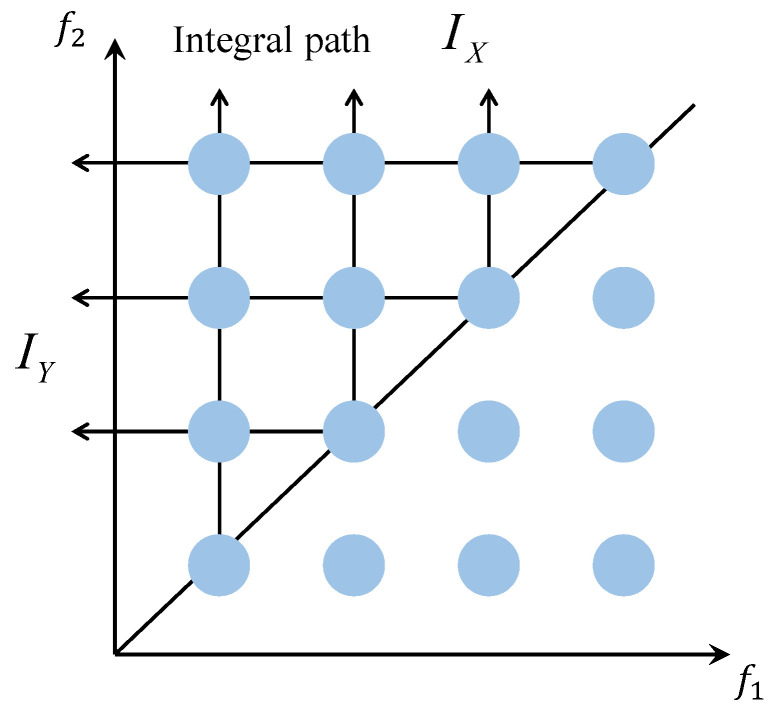
DBIB schematic diagram.

As illustrated in Figure 5, based on the specific representation of the harmonic components within the signal in the bispectrum matrix, we propose a bispectrum integral path known as the Diagonal Bi-Directional Integral Bispectrum (DBIB), which is suitable for harmonic feature extraction. The horizontal and vertical axes are integrated separately along the diagonal of the first quadrant. This method preserves the frequency coupling characteristics of harmonics, and through the integration results $I_{X}$ and $I_{Y}$, the harmonic characteristics can be analyzed in greater depth.

In the bispectral matrix, due to its symmetry, analyzing the first quadrant ($f_{1}>0$, $f_{2}>0$) alone is sufficient to represent all the information. By dividing the matrix along $f_{1}=f_{2}$ (the 45° diagonal), the upper triangular region, where $f_{1}<f_{2}$, is selected. This approach eliminates redundancy in the bispectral $\left(f_{1},f_{2}\right)$ pairs and ensures that each pair is unique.

(6) $$I_{x}\left(f_{1}\right)=\sum_{f_{2}\;>\;f_{1}}B\left(f_{1},f_{2}\right)$$

Specifically, for each $f_{1}$, the bispectral values corresponding to all $f_{2}>f_{1}$ are summed, which reflects the strength of the non-linear (harmonic) interactions between $f_{1}$ and all higher-frequency components.

(7) $$I_{y}\left(f_{2}\right)=\sum_{f_{1}\;<\;f_{2}}B\left(f_{1},f_{2}\right)$$

Specifically, for each $f_{2}$, the bispectral values corresponding to all $f_{1}<f_{2}$ are summed, which reflects the strength of the non-linear (harmonic) interactions between $f_{2}$ and all higher-frequency components.

$I_{x}$ represents the total strength of non-linear coupling between each $f_{1}$ and all higher-frequency $f_{2}$, highlighting the importance of $f_{1}$ as the fundamental frequency participating in non-linear interactions. $I_{y}$ represents the total strength of non-linear coupling between each $f_{2}$ and all lower-frequency $f_{1}$, emphasizing the significance of $f_{2}$ as the target frequency being synthesized. In other words, $I_{x}$ and $I_{y}$ describe the distribution of each frequency’s role as the initiating and target frequency, respectively, throughout the entire non-linear harmonic process.

**Figure 6:**
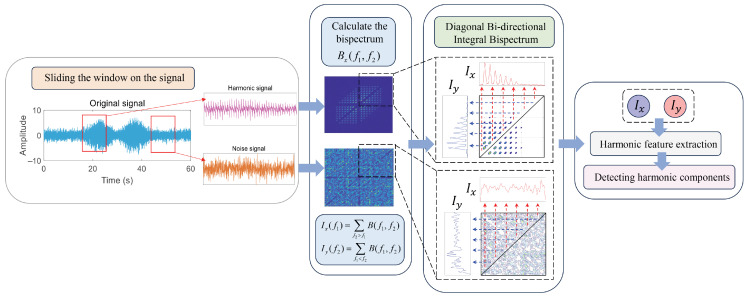
DBIB calculation flowchart.

The specific workflow of the Diagonal Bi-Directional Integral Bispectrum (DBIB) in signal processing is illustrated in Figure 6. The signal to be analyzed is divided into windows, and the signal fragment extracted from each window is processed using the bispectrum. If a harmonic component is present in the signal, it will be represented in the bispectrum matrix. Beginning from the points on the diagonal, integration is performed along the vertical and horizontal axes to obtain ($I_{x}$) and ($I_{y}$). The results indicate a strong frequency coupling characteristic of harmonics, primarily due to the strong correlation of harmonic frequency doubling with the fundamental frequency, whereas background noise lacks this characteristic, and this facilitates the subsequent harmonic detection task.

### 2.3. Harmonic Aggregation Entropy

Based on the Diagonal Bi-Directional Integral Bispectrum (DBIB), accurately assessing the strength of harmonic components from the relative relationship between $I_{X}$ and $I_{Y}$ alone proves challenging. In this context, we propose a new metric known as harmonic aggregation entropy to quantify the harmonic strength of time series [36,37]. The essence of this metric lies in calculating the mode difference between $I_{X}$ and $I_{Y}$, with the core concept being the quantification of the probability that the harmonic frequency band is present in both integration results. Prior to the calculation of harmonic aggregation entropy (HaAgEn), we define the embedding dimension *m* and the threshold *r*. Here, *m* is a positive integer greater than 0, typically set to 2 or 3 [38,39], while *r* is related to the standard deviation σ of the data [40] and is usually defined as follows:

(8) r=0.1∼0.25×σ

Upon establishing the parameters, we proceed to construct the embedding vector:

(9) $$I_{x}^{m}\left(i\right)=\left[I_{x}\left(i\right),I_{x}\left(i+1\right),\ldots ,I_{x}\left(i+m-1\right)\right]\;\left(1\leq i\leq N-m+1\right)$$

(10) $$I_{y}^{m}\left(j\right)=\left[I_{y}\left(j\right),I_{y}\left(j+1\right),\ldots ,I_{y}\left(j+m-1\right)\right]\;\left(1\leq j\leq N-m+1\right)$$

Next, compute the cross-sequence vector distance:

(11) $$d\left(I_{x}^{m}\left(i\right),I_{y}^{m}\left(j\right)\right)=\max_{0\leq k\leq m-1}\left|I_{x}\left(i+k\right)-I_{y}\left(j+k\right)\right|\leq r$$

For each vector $I_{x}^{m}\left(i\right)$, count the number of vectors that satisfy the condition $d(I_{x}^{m}\left(i\right)$, $I_{y}^{m}\left(j\right))\leq r$, which is denoted as $C_{i}^{m}\left(r\right)$.

(12) $$C_{i}^{m}\left(r\right)=\frac{\sum_{j=1}^{N-m+1}1_{\left\{d\left(I_{x}^{m}\left(i\right),I_{y}^{m}\left(j\right)\right)\leq r\right\}}}{N-m+1}$$

Calculate the average logarithmic probability:

(13) $$\Phi^{m}\left(r\right)=\frac{1}{N-m+1}\sum_{i=1}^{N-m+1}\ln C_{i}^{m}\left(r\right)$$

Ultimately, the HaAgEn can be computed using the following equation:

(14) $$\mathrm{HaAgEn}\left(m,r,N\right)=\Phi^{m}\left(r\right)-\Phi^{m+1}\left(r\right)$$

**Figure 7:**
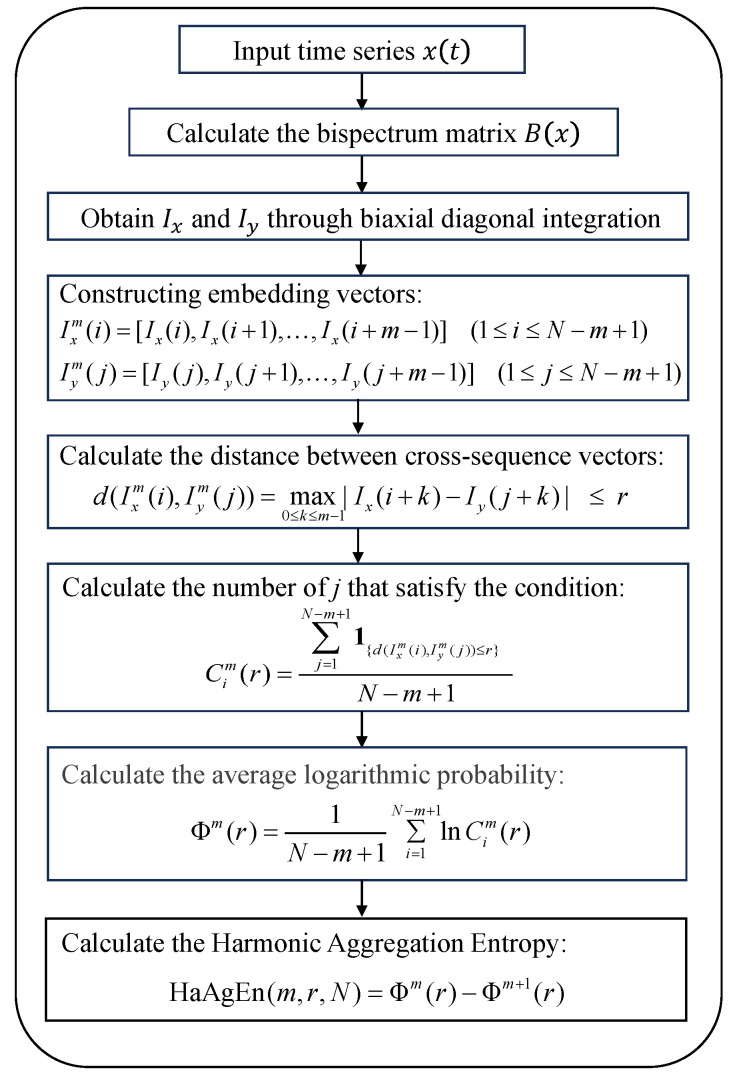
HaAgEn calculation flowchart.

The comprehensive calculation process of HaAgEn, derived from DBIB, is illustrated in Figure 7. This method enhances the simplicity of the results obtained from bispectral integration and accurately represents the harmonic characteristics through a highly representative entropy value, thereby significantly reducing the computational effort required for harmonic detection.

## 3. Simulation Analysis

**Figure 8:**
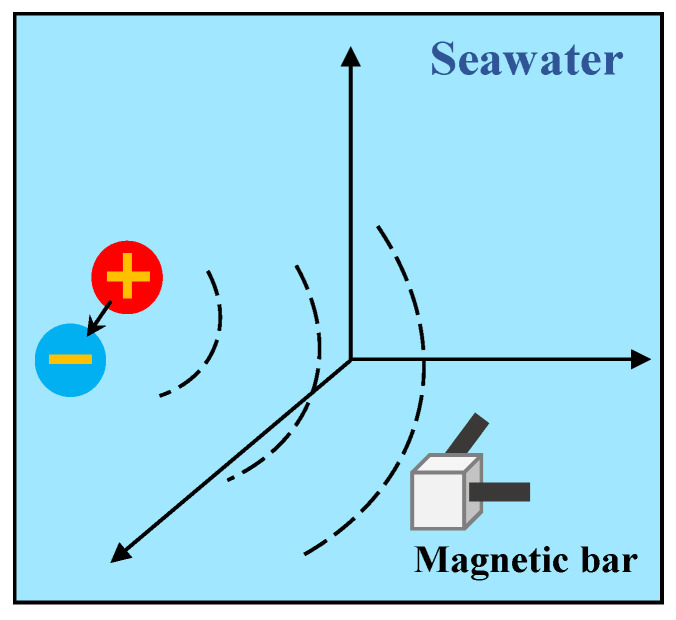
Schematic diagram of shaft-rate magnetic field signal acquisition.

To validate the effectiveness of HaAgEn in characterizing harmonic features, we simulated the outward propagation of the magnetic component signal generated by a current element (analog electric dipole) in seawater for analytical purposes. Typically, a pair of orthogonal magnetic rods is employed underwater to measure the axial frequency magnetic field signal of the target, as depicted in Figure 8. This method allowed us to assess the presence of electromagnetic leakage in the target by detecting the harmonic component within the signal.

**Figure 9:**
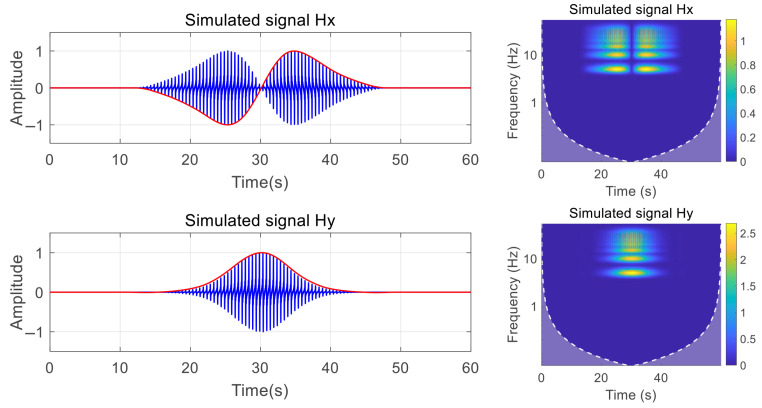
Magnetic field component signal time–frequency diagram.

In this context, we simulated the axial frequency magnetic field signal featuring a fundamental frequency of 5 Hz and its eighth harmonic. The Hx and Hy components of the magnetic field signal, along with their continuous wavelet transform time–frequency diagram, are presented in Figure 9.

**Figure 10:**
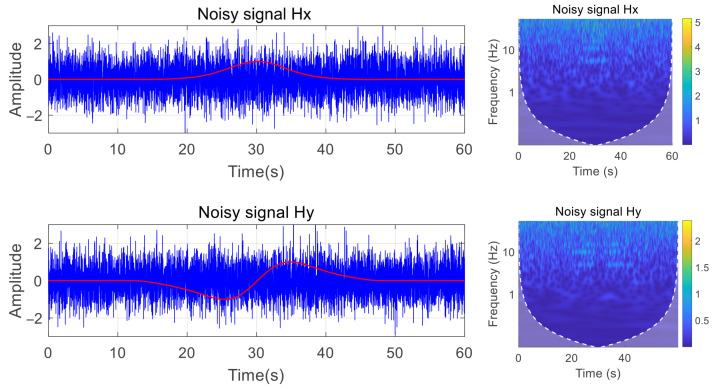
Magnetic field components and time–frequency diagrams at SNR of −5.

Gaussian white noise with an SNR of −5 was added to the simulation signal. As illustrated in Figure 10, extracting the harmonic features of the signal through time–frequency analysis under this SNR is challenging, and only the fundamental frequency component at 5 Hz is faintly observable. Consequently, distinguishing the harmonic signal from background noise will be challenging if only the time–frequency image is input into the convolutional neural network for detection.

**Figure 11:**
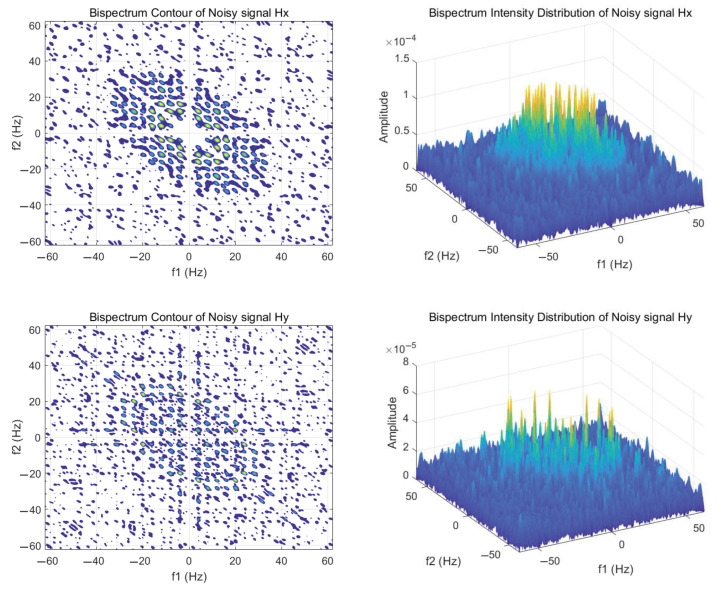
Bispectrum contour plot and its intensity distribution.

To demonstrate that conventional time–frequency analysis struggles to extract harmonic features from signals with low SNR, we utilize bispectrum analysis to extract harmonic components from the signal. As illustrated in Figure 11, the bispectrum contour plot and intensity distribution of the Hx and Hy components of the axial frequency magnetic field signal with an SNR of −5 are presented.

It is evident that, in comparison to time–frequency analysis, bispectrum analysis exhibits greater sensitivity to the harmonic characteristics of signals, particularly the frequency coupling characteristics, and clearly illustrates the correlation between the fundamental frequency and each harmonic order. When $f_{2}$ is set to 5 Hz in the figure, the periodic bright spots that appear as $f_{1}$ increases represent the harmonic signal with a 5 Hz fundamental frequency, which clearly distinguishes itself from the surrounding noise. However, directly using the bispectral matrix as the harmonic feature input involves a significant computational load, making it unsuitable for machine learning applications. To reduce the dimensionality of harmonic features, we propose a novel integration method for harmonic features.

Furthermore, we introduce a new index to evaluate the strength of signal harmonic components: Harmonic Aggregation Entropy. The calculation process of this entropy emphasizes the frequency coupling characteristics of harmonics, resulting in a strong consistency between $I_{X}$ and $I_{Y}$ in the bispectrum integration results, thereby reflecting the periodicity and correlation of harmonic components.

**Figure 12:**
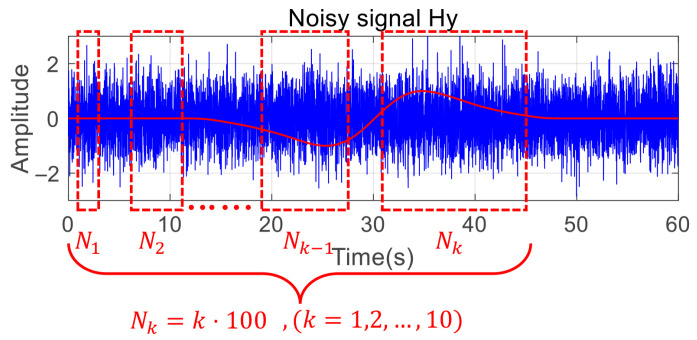
Divide computation windows of different sizes.

During the calculation of HaAgEn, the window size *N* is a critical factor. If the window size is small, the harmonic features may be inadequately calculated, resulting in ambiguous features. Conversely, if the window size is too large, excessive noise may be introduced into the analysis. Therefore, it is essential to analyze and determine the optimal window size *N*, and we design several different window sizes, as illustrated in Figure 12.

Given that the positions of harmonics in the simulation signal are known, we compute the difference between the average HaAgEn values in the noise and harmonic intervals of the calculation results to quantify the entropy difference between the harmonic and noise components. A larger difference indicates a more favorable condition for detection and a more pronounced effect of entropy. The calculation is as follows:

(15) $$\Delta E_{n}=\bar{{\mathrm{HaAgEn}}_{\mathrm{Noise}}}-\bar{{\mathrm{HaAgEn}}_{\mathrm{Signal}}}$$

**Figure 13:**
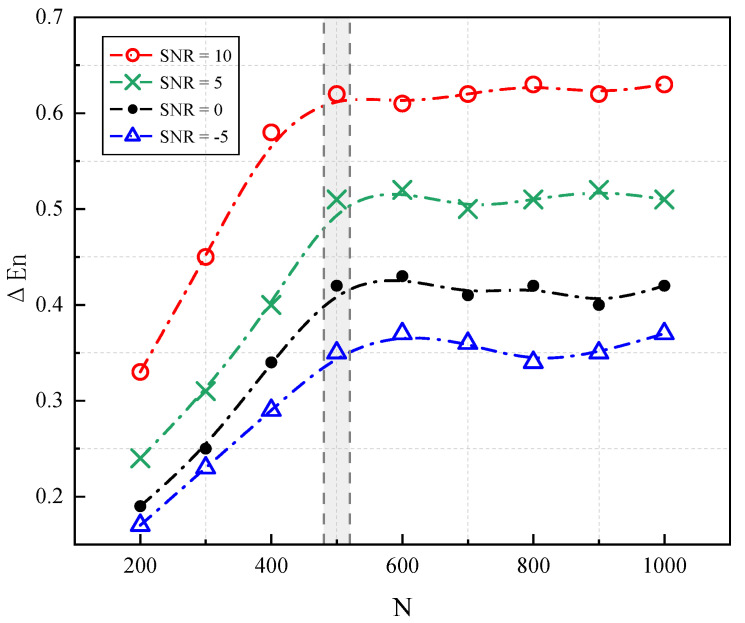
The $\Delta E_{n}$ corresponding to different window sizes at various signal-to-noise ratios.

The calculated $\Delta E_{n}$ at different signal-to-noise ratios and various window sizes N is shown in Figure 13. By comparing the $\Delta E_{n}$ of the HaAgEn for signal and noise segments under different window sizes, it can be seen that as N increases, the difference in the entropy value between the noise and harmonic signals gradually increases. After N reaches 500, $\Delta E_{n}$ stabilizes, indicating that the window size is sufficient for the characteristic extraction of harmonic components and noise by harmonic aggregation entropy. Further increasing the window size does not significantly improve the extraction effect but adds extra computational load. Therefore, a window size of 500 will be used in subsequent experiments in this study.

**Figure 14:**
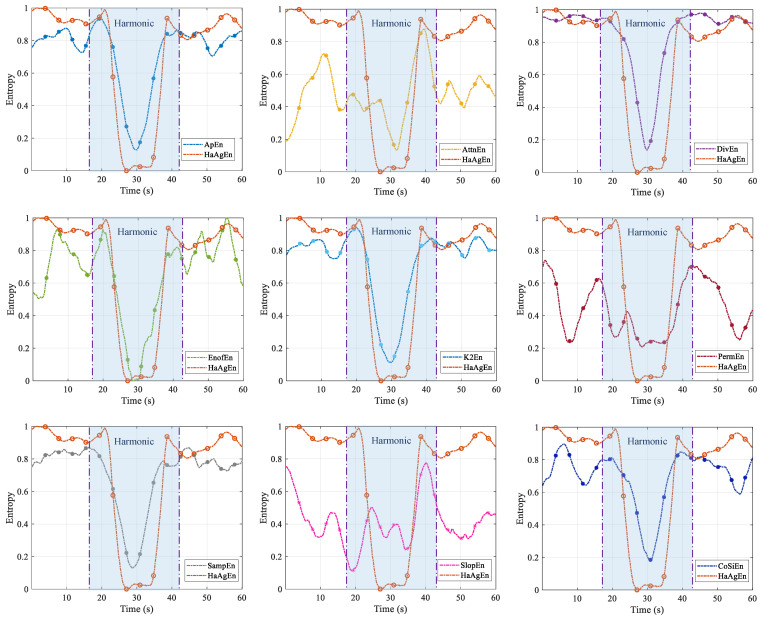
Sensitivity analysis of different entropy and HaAgEn to signal harmonic components.

Building on this foundation, we further investigate the advantages of the proposed entropy method for harmonic feature extraction. We select a range of entropy measures that are currently employed to assess the complexity of time series for comparison, with the results illustrated in Figure 14. The regularity of harmonics elicits varying responses from these entropy measures. This approach eliminates the numerical differences between the various entropy measures, resulting in a standardized metric that quantifies the difference between the entropy values of the noise and harmonic intervals, denoted as γ:

(16) $$\gamma =\frac{\Delta E_{n}}{En_{\mathrm{Noise}}}=1-\frac{\bar{En_{\mathrm{Signal}}}}{\bar{En_{\mathrm{Noise}}}}$$

The discrimination degree γ of noise and harmonics corresponding to different entropy measures is presented in Table 1. It is evident that HaAgEn outperforms other entropy types significantly, particularly in the harmonic interval, where the entropy value remains relatively stable. This aligns with the spectral characteristics of the signal within this interval and demonstrates that HaAgEn exhibits strong robustness in characterizing the harmonic strength of the signal.

## 4. Sea Trial Data Validation

**Figure 15:**
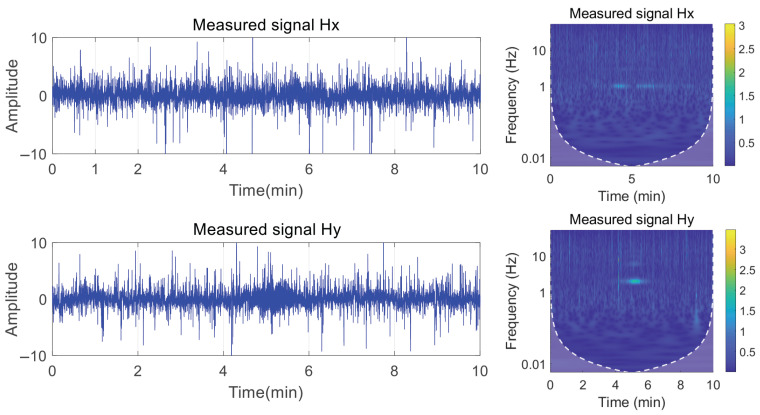
Measured shaft-rate magnetic field signal and marine electromagnetic noise.

To verify whether the proposed method is effective for measuring harmonic components in actual signals, we selected sea trial data from June 2024 for analysis. The experiment used a towed electrode transmitter to emit extremely low-frequency electromagnetic signals into the seawater to simulate axial-frequency electromagnetic field signals. These extremely low-frequency electromagnetic signals, embedded in real marine electromagnetic noise, were measured using orthogonal magnetic sensors arranged underwater. This signal contains typical harmonic characteristics of power systems. The actual measured signal is shown in Figure 15:

The purpose of this paper is to validate that the proposed method has better performance in detecting harmonic components of signals compared to traditional time–frequency analysis methods. Therefore, the most basic CNN network is chosen for testing, which consists of seven layers: input layer, convolution layers (C1 and C2), pooling layers (S1 and S2), fully connected layer (F1), and output layer. First, using a 1D-CNN with only the original time series as input, the effectiveness of different bispectral integration methods for harmonic detection is tested. Then, a 2D-CNN is used to examine the improvement in harmonic signal detection accuracy by adding HaAgEn features to bispectral images with different two-dimensional image inputs.

**Figure 16:**
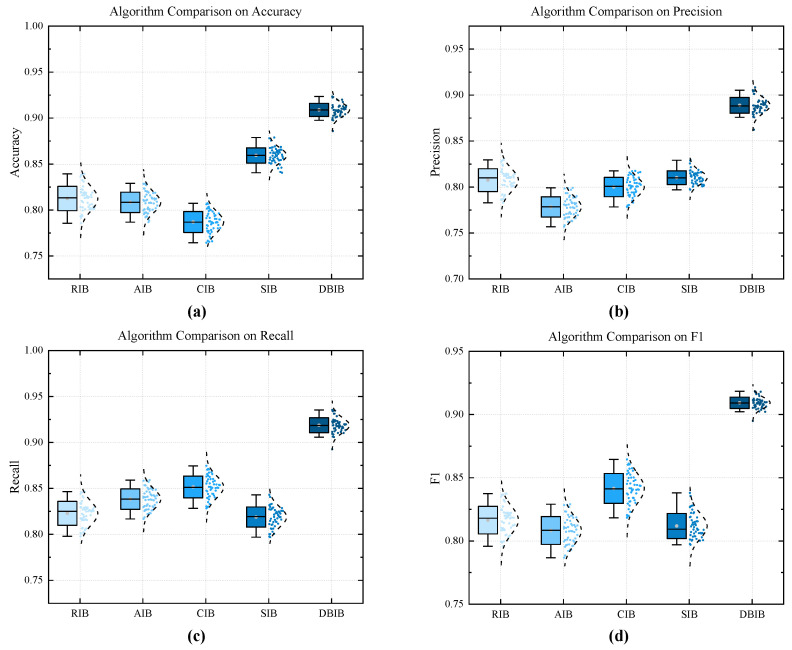
Performance differences of five bispectrum integration methods for detecting harmonics: (**a**) Accuracy. (**b**) Precision. (**c**) Recall. (**d**) F1.

**Figure 17:**
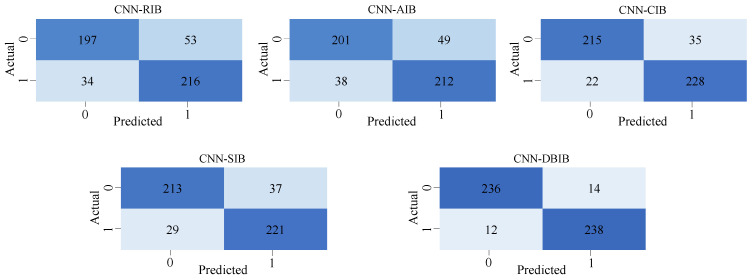
Confusion matrices for five bispectrum integration methods for detecting harmonics.

The sea trial dataset was divided into training and testing sets in an 8:2 ratio. The accuracy, recall, F1 score, and precision of each method were evaluated and compared. The corresponding results are presented in Figure 16. First, a comparative analysis of the performance of five bispectral integration methods on CNN was conducted. As seen in the Figure 17, DBIB’s data surpasses other integration methods in all aspects, with the recognition accuracy reaching 94.8%. This proves that our proposed integration method has a very significant effect on the extraction of harmonic features from signals.

**Figure 18:**
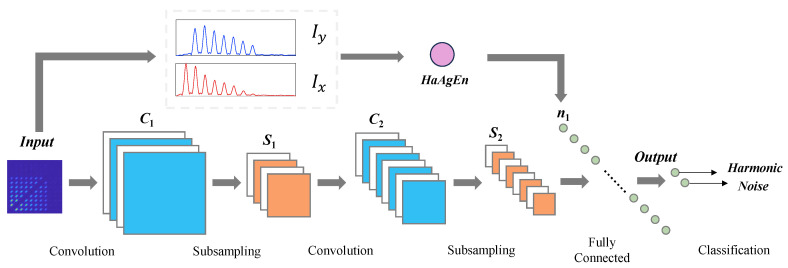
Network structure of 2D-CNN with added HaAgEn features.

**Figure 19:**
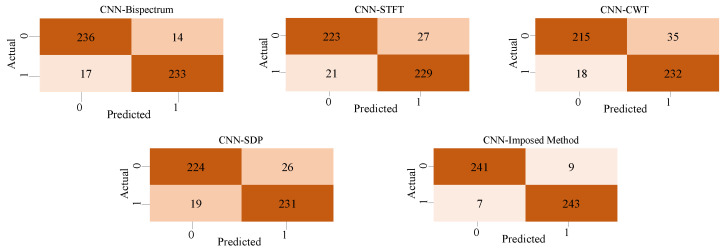
Confusion matrix of four 2D image harmonic detection methods.

On this basis, we further studied the improvement in harmonic signal detection capability when HaAgEn is combined with the bispectral matrix. Similar to the traditional method of inputting time–frequency graphs into convolutional neural networks, we input bispectral graphs along with STFT, CWT, and SDP time series of 2D images into the CNN for training. However, while inputting bispectral graphs, we also calculate HaAgEn as an additional feature to be input into the fully connected layer. This is to test its effectiveness in harmonic feature detection. The CNN model structure with added entropy features is shown in the Figure 18. Using this model, we trained each of the five methods and conducted 100 tests on the test set. The average detection results are shown in Figure 19.

**Figure 20:**
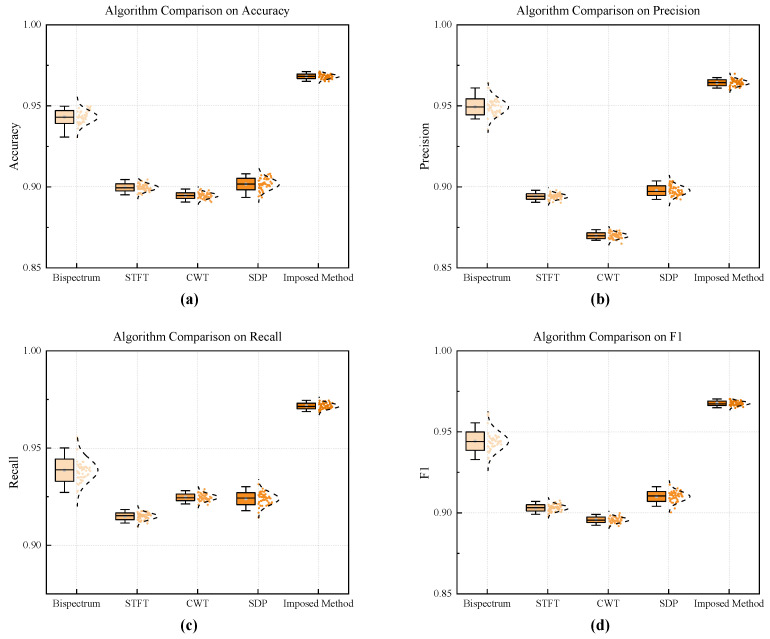
Performance differences of four harmonic detection methods for 2D images: (**a**) Accuracy. (**b**) Precision. (**c**) Recall. (**d**) F1.

By comparing several 2D time series transformation methods in detecting harmonic components of shaft-frequency magnetic signals using CNN across four key indicators—accuracy, precision, recall, and F1 score—it can be found that the method proposed in this paper significantly outperforms other methods in terms of average performance on all indicators. From the distribution of box plots, it can be seen that the performance fluctuations of the method proposed in this paper are almost negligible. As shown in Figure 20 and Table 2, this method significantly surpasses other approaches in terms of both robustness and detection accuracy, with the accuracy rate reaching 96.8%.

Although traditional STFT, CWT, and SDP methods can transform time series data into 2D images, they only reflect the time-domain and frequency-domain characteristics of the signal in the 2D images, and do not clearly capture the frequency coupling characteristics in harmonics. This paper, based on the bispectral matrix, incorporates HaAgEn features at the fully connected layer, which not only retains the 2D image characteristics of the harmonic bispectrum but also adds entropy features as auxiliary features, achieving a notable enhancement in detection performance.

## 5. Conclusions

HaAgEn, as a novel feature estimation approach for harmonic features in time series, has been proven effective on both simulated data and actual maritime measurement data. Its particular innovations and advantages are as follows:

(1) To address the issue where existing bispectral integration methods cannot effectively extract harmonic features from time series, an innovative use of the frequency coupling characteristics of harmonic signals in the bispectral matrix was employed, leading to the proposal of the DBIB integration method. This method significantly outperformed other integration methods in comparative experiments and served as a foundation for further developing HaAgEn.
(2) To address the situation where various types of entropy values cannot effectively characterize harmonic features in signals, further calculations were performed on the bispectral integration results based on DBIB, resulting in the HaAgEn of the time series. This was compared with various other types of entropy in experiments, demonstrating that its sensitivity to harmonics is significantly superior to other methods.
(3) By integrating HaAgEn with convolutional neural networks, the method proposed in this paper significantly outperforms traditional methods of detecting harmonics using time–frequency feature inputs, such as STFT and CWT, especially within real maritime measurement datasets, achieving a detection accuracy of 96.8%.

HaAgEn combines bispectral integration with entropy theory to effectively quantify the aggregation level and complexity of harmonic components, thereby achieving high-discrimination feature extraction for target signals. The research findings of this paper provide new insights into electromagnetic signal analysis in underwater detection and ship safety protection fields, and also offer new technological approaches for harmonic detection in power systems and industrial fields.

## Figures and Tables

**Table 1:** Entropy and γ values.

Entropy	γ	Entropy	γ
ApEn	0.37	PermEn	0.28
ApEn	0.21	SampEn	0.35
DivEn	0.37	SlopeEn	0.18
EnofEn	0.36	CoSiEn	0.36
K2En	0.38	HaAgEn	0.43

**Table 2:** Performance comparison of different methods.

Method	Accuracy	Precision	Recall	F1
CNN-Bispectrum	0.938	0.943	0.932	0.938
CNN-STFT	0.904	0.895	0.916	0.905
CNN-CWT	0.894	0.869	0.928	0.897
CNN-SDP	0.91	0.90	0.924	0.911
CNN-Imposed Method	0.968	0.964	0.972	0.968

## Data Availability

The data that support the findings of this study are available from the corresponding author, [D.W.], upon reasonable request.
